# Surgical management of a huge post-circumcision epidermoid cyst of the vulva presenting unusually in a postmenopausal woman: a case report

**DOI:** 10.1186/s13256-018-1773-1

**Published:** 2018-08-23

**Authors:** Wondimu Gudu

**Affiliations:** Department of Obstetrics and Gynecology, Saint Paul’s Hospital Millennium Medical College, P.O. Box 1271, Addis Ababa, Ethiopia

**Keywords:** Epidermoid, Cyst, Female genital mutilation, Vulva, Surgery

## Abstract

**Background:**

Epidermoid cysts of the external genitalia are one of the late complications of female genital mutilation. These cysts are usually small and painless. The presentation of a giant vulvar cyst in a postmenopausal woman has not been reported; the surgical management of a large cyst is often challenging.

**Case presentation:**

A 60-year-old, para VI, Ethiopian woman presented with an 18 cm by 12 cm sized, fluctuant, multi-lobulated, mobile, non-tender vulvar mass involving the mons pubis. She had undergone genital cutting and sewing during her childhood. Informed consent was obtained for surgery and the vulvar cyst was excised successfully without any significant bleeding or injury to the adjacent structures. Her postoperative course was smooth and histopathology confirmed epidermoid cyst of the vulva.

**Conclusions:**

Huge epidermoid vulvar cysts following genital mutilation are rare and presentation in a postmenopausal woman has not been reported. Practitioners in areas where female genital mutilation is prevalent should be aware of the peculiar anatomic and technical aspects of the surgical removal of these giant vulvar cysts for successful therapeutic and cosmetic outcomes.

## Background

Epidermoid cysts of the external genitalia (vulva) are one of the late complications of female genital mutilation (FGM). These cysts arise as a result of invagination/implantation of squamous epithelium under the dermis or subcutaneous tissue during the procedure of FGM which leads to the accumulation of epidermal desquamations, secretions, and other debris in a closed space [1, 2].

Epidermoid cysts of the vulva are often painless and small in size and often present in perimenarcheal women. The diagnosis is usually made by clinical examination. Ultrasonography (US) and magnetic resonance imaging (MRI) are helpful in differentiating epidermoid cysts from other vulvar tumors [3, 4].

The management of vulvar cysts is excision. However, the surgical removal of a large cyst is challenging [3]. Here, we have discussed the first case of a huge post-circumcision epidermoid cyst of the vulva in a postmenopausal woman who was managed surgically with successful clinical and cosmetic outcomes. The case is peculiar because the presentation of huge vulvar epidermoid cysts postmenopause is extremely rare. Hence it will give important insights to clinicians in the surgical management of such a very rare vulvar disorder.

## Case presentation

A 60-year-old, para VI, Ethiopian woman presented with a progressively increasing vulvar swelling of 25 years’ duration. She developed a dull aching pain 3 months previously with difficulty of micturition and dysuria. There was no discharge or bleeding. She had undergone genital cutting and sewing during her childhood. Her medical and surgical histories were unremarkable. She is unemployed. There was no similar history of illness in her family. She neither smokes tobacco nor drinks alcohol. She had not visited any other health care facility for the complaint and had not been on any medication prior to diagnosis.

On physical examination, her vital signs were: pulse, 82; respiratory rate, 18; and temperature, 36 °C.

**Fig. 1:**
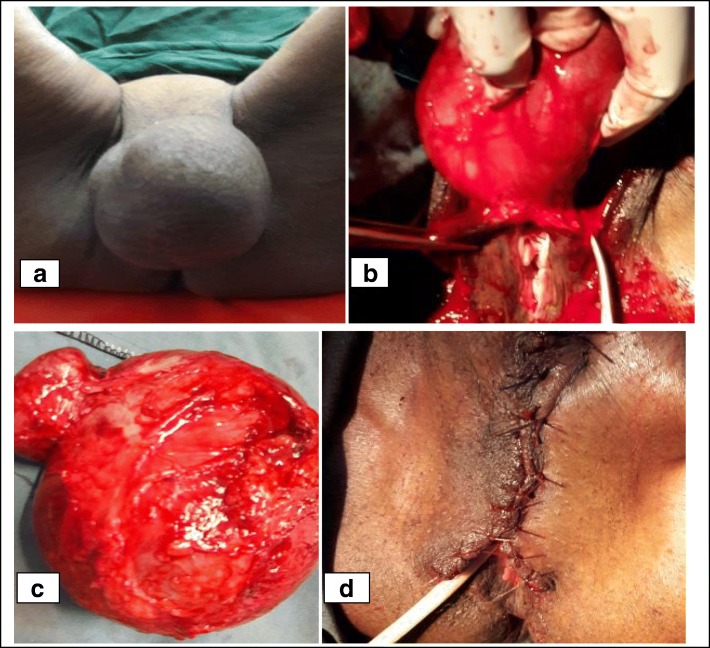
Clinical images during the surgical excision of the huge vulvar cyst. **a** The appearance of the huge vulvar cyst before surgery. Note that it has extended to the mons pubis and obscured the urethral meatus. **b** The cyst is dissected off the overlying skin. The base of the cyst is seen incorporating ventral skin of the labia minora and stretching urethra meatus. Care should be taken not to injure the labia minora and urethra. **c** The appearance of the multi-lobulated cyst after complete excision. **d** The vulva after layered closure of the incision site. Note that the two labial edges are sutured separately and urinary catheter is inserted

She had pink conjunctiva and non-icteric sclera; her chest was clear and resonant. Her heart sounds were all normal with no murmur or gallop. Her abdomen was soft and moved with respiration. There was no tenderness, guarding rigidity, palpable mass, or organomegaly. In her genitourinary system, there was a 18 cm by 12 cm sized, fluctuant, multi-lobulated, mobile, non-tender, right labia majora mass involving the mons pubis and stretching the ventral skin of the urethra (Fig. 1a). The overlying skin was normal. The two labial edges were fused at their cranial part. There were no abnormal findings in her vagina, cervix, and uterus on speculum and digital examinations. The inguinal lymph nodes were not enlarged.

Musculoskeletal and neurological examinations were unremarkable.

Laboratory tests results were: hemoglobin, 13 gm/dl; white blood cell count, 6500/mm^3^; platelet count, 250,000; and blood group/Rh B^+^. Urine analysis was non-revealing; a pelvic ultrasound scan showed atrophied uterus with no pelvic mass.

She was counseled for surgical removal (excision) and informed consent was obtained. Under spinal anesthesia, a urinary Foley catheter was inserted; the vulvar mass was excised successfully without any significant bleeding or injury to the adjacent structures. Her postoperative course was smooth with healed wound site on postoperative day 7 checkup visit. She had her second postoperative visit 1 month after the surgery and she had no genitourinary complaints with healed vulvar wound site. A histopathologic examination report was consistent with epidermoid cyst of the vulva.

## Discussion

This case report described an extremely rare presentation of a huge post-circumcision epidermoid cyst of the vulva in a postmenopausal woman who was successfully managed surgically with optimal clinical and cosmetic outcomes.

Although cases of large clitoral epidermoid inclusion cysts following genital mutilation have been reported in young women [5, 6], this is the first case of a vulvar cyst in a postmenopausal woman and the vulvar cyst is the biggest reported in the literature.

The occurrence of epidermoid cyst is an expected complication in our patient who had type II/III FGM during childhood. However, it is unusual for the cysts to attain such a very big size in the postmenopausal period. A rapid growth of the cysts is usually expected in perimenarcheal women in whom there is an increase in vulval and vaginal secretions secondary to the high levels of estrogen, which invariably leads to an increase in the secretion of the epidermal cells in the inner lining of the cyst wall that are of vulval origin [2].

Epidermoid cysts are usually slow growing over years [3]. Hence, most patients present late unless they develop symptoms of complications like pain, discharge from the swelling, and difficulty of urination or walking. The fact that our patient presented late despite having such a very big vulvar swelling might be due to her poor health-seeking behavior or lack of knowledge of availability of medical services. However, importantly, it could be a reflection of the lack of integrated services for the management of FGM complications in the nearby health facilities where FGM is highly prevalent.

The clinical diagnosis is usually made by careful genital examination. US can differentiate a cystic mass and its relationship with clitoris and urinary tract. MRI is important to characterize the location and consistency of the vulvar mass and its extension to surrounding tissue [3, 4]. A histopathologic examination will confirm the diagnosis. The differential diagnoses include any cystic vulvar lesions including Bartholin cyst, epidermoid cyst, Gartner’s cyst, cyst of canal of Nuck, lipoma, endometrioma, posttraumatic hematoma, and inguinal hernia [4, 7].

The management of large dermoid cysts is usually challenging and deserves special considerations because of the associated perioperative complications. The labia minora and urethra/external urethral meatus are usually incorporated and stretched over their ventral proximal aspect by the giant cysts which predispose the structures for surgical injury [2, 4]. In addition, if the cysts extend to the clitoris or deep into perineal membrane, there is a risk of heavy bleeding from injury to the clitoral or inferior hemorrhoidal branches of the pudendal vessels [8].

The major anatomical and technical considerations to ensure optimal clinical and cosmetic outcomes during the surgical removal of giant epidermoid cysts which were performed in our case are highlighted below.

1. A preoperative revision of the anatomy of the external genitalia is helpful. Identification of the proper dissection plane will make an easy separation of the cyst intact from underlying structures. In addition, avoiding inadvertent incision of the cyst is important as keeping the cyst intact will make dissection easier. This inadvertent pricking/incision will be prevented by carefully observing the overlying skin and noting puckered/scarred/indented areas, which are markers of a previous breach in the integrity of the skin, and by avoiding deep dissection at these sites.
2. Meticulous hemostasis including the identification and ligation of the clitoral and inferior hemorrhoidal branches of the pudendal vessels will prevent unnecessary blood loss [8] and decrease the risk of postoperative hematoma collection and subsequent wound failure.
3. Identification and catheterization of the urethral meatus is necessary to avoid injury during dissection as well as to avoid unintentional suturing of the urethral meatus/suburethral tissue during closure of the cyst bed (Fig. 1b).
4. Complete excision of the cyst ensuring that the base of the cyst is entirely removed is important to avoid the risk of recurrence from leftover epidermal tissue at the base of the cyst. In the case of clitoral cysts, an attempt to preserve clitoral tissue buried under the scar of the fused vulvar edges has been advised [9, 10]. However, it is reported that the risk of recurrence is high if the clitoris is not entirely removed [2, 5, 6, 11]. Hence, recently the complete removal of the clitoris (total clitoridectomy) with preservation of the ventral skin of clitoris and labia minora is advised in young patients. The preservation of these tissues is reported to be enough to ensure that women will achieve orgasm and experience sexual satisfaction [12, 13]. Since our case was in menopause, total excision of the cyst including removal of clitoral tissue was done.
5. Approximation of the subcutaneous fasciae of the mons and the labia majora will facilitate easy closure of the skin edges without tension and will decrease the risk of subcutaneous hematomas (Fig. 1d).
6. Leaving a glove or Penrose drain if a large dead space is left is wise.

## Conclusions

A huge post-circumcision epidermoid cyst of the vulva is extremely rare and its presentation in a postmenopausal woman has not been reported. These vulvar epidermoid cysts might be easily misdiagnosed for other neoplastic vulvar disorders by inexperienced clinicians. Unlike small cysts, the surgical excision of these cysts is associated with substantial perioperative complications. Practitioners in FGM-prevalent areas should be aware of the peculiar anatomic and technical aspects of the surgical procedure for successful therapeutic and cosmetic outcomes.

## Abbreviations

FGM: Female genital mutilation
MRI: Magnetic resonance imaging
US: Ultrasonography
